# Analysis of change in gait in the ovine stifle: normal, injured, and anterior cruciate ligament reconstructed

**DOI:** 10.1186/s12891-017-1576-3

**Published:** 2017-05-23

**Authors:** B. J. Heard, J. E. Beveridge, M. Atarod, E. J. O’Brien, C. Rolian, C. B. Frank, D. A. Hart, N. G. Shrive

**Affiliations:** 1 The McCaig Institute for Bone and Joint Health, University of Calgary, Calgary, AB Canada; 2 Department of Civil Engineering, University of Calgary, Calgary, AB Canada; 3 Department of Comparative Biology and Experimental Medicine, Faculty of Veterinary Medicine, University of Calgary, Calgary, AB Canada

**Keywords:** Ovine, Principal Component Analysis, Kinematics, ACL reconstruction, Simplified

## Abstract

**Background:**

Many patients who undergo anterior cruciate ligament (ACL) reconstructive surgery develop post-traumatic osteoarthritis (PTOA). ACL reconstructive surgery may not fully restore pre-injury joint biomechanics, thereby resulting in further joint damage and contributing to the development of PTOA. In an ovine model of idealized ACL reconstruction (ACL-R), it has been shown that signs of PTOA develop within surgical joints by 20 weeks post-surgery. The aim of the present study was to investigate whether altered kinematics contribute to early PTOA development within ACL-R joints of the ovine injury model by comparing the gait of these surgical animals to the gait of a stable normal control group, and an unstable injury group in which the ACL and medial collateral ligament (MCL) had been transected.

**Methods:**

Fifteen skeletally mature female sheep were allocated evenly into 3 treatment groups: normal control, ACL-R, and ACL/MCL Tx (each group *n* = 5). Each animal’s gait was recorded at baseline, 4 weeks post injury, and 20 weeks post injury. Principal component analysis (PCA) was used to identify the kinematic patterns that may be discriminant between treatment groups. Results from previous studies were referenced to present the amount of gross PTOA-like changes that occurred in the joints.

**Results:**

ACL-R and ACL/MCL transected (Tx) animals developed a similar amount of early PTOA-like changes within the surgical joints, but differed significantly in the amount of kinematic change present at 20 weeks post-surgery. We showed that the stifle joint kinematics of ACL/MCL Tx differed significantly from those of CTRL and the majority of ACL-R animals, while no significant differences in joint kinematic changes were found between ACL-R and CTRL animals.

**Conclusions:**

These results suggest that the early PTOA-like changes reported in the ACL-R model cannot be attributed exclusively to post-surgical kinematic changes, and therefore biologic components in the post-injury environment must be contributing significantly to PTOA development.

**Electronic supplementary material:**

The online version of this article (doi:10.1186/s12891-017-1576-3) contains supplementary material, which is available to authorized users.

## Background

Poor long-term clinical outcomes are associated with anterior cruciate ligament (ACL) injuries, with up to 50% of patients developing post-traumatic osteoarthritis (PTOA) by 20 years after injury [1]. Despite restoring joint stability subjectively, ACL reconstructive surgery does not prevent the onset of PTOA [2]. Thus all ACL-reconstructed patients are at risk of developing PTOA, and develop the disease at a younger age than patients who suffer from idiopathic OA [3]. The inability to prevent secondary degenerative changes represents an enormous economic burden on health care systems [4, 5], which is compounded by OA-associated co-morbidities such as depression [6] and increased risk of cardiovascular disease [7]. Thus, there is an urgent need to develop an understanding of PTOA pathogenesis following ACL injury and reconstruction in order to develop efficient interventions that prevent the onset of PTOA and improve patient outcomes.

Our research group and others [8] have studied large animal models of ACL injury and reconstruction extensively in an effort to reveal the interplay between biological and mechanical changes that drive long-term joint damage. Our ovine model of combined ACL and medial collateral ligament transection (ACL/MCL Tx) recapitulates the individual variability in gross morphological damage observed in human patients [9]. We have shown that the variability in damage is associated with the degree of joint motion abnormality, specifically increases in anterior and medial tibial translation 20 weeks post-transection [10, 11]. Furthermore, abnormal surface motions that are a function of all 6° of freedom (DOFs), also correlate with cartilage damage [12]. We have also developed a unique ACL reconstruction model (ACL-R) that we believe maintains joint stability while simultaneously creating “ideal” ACL graft properties by using the native ACL as the graft material and fixing the “graft” in its original anatomic position [13, 14]. While the idealized ACL-R appears to maintain stifle stability, we noted significant early PTOA-like morphological changes, as quantified by the prevalence of osteophyte formation and cartilage damage, compared to non-operated control and surgical sham (arthrotomy only) stifles [15]. The magnitude of early PTOA-like changes differs significantly between ACL-R and sham stifles, whereas the magnitude of post-operative kinematic changes in these two models over time are nearly comparable in a pooled group analysis [13]. Conversely, the magnitude of early PTOA-like gross morphological changes that occur in both ACL/MCL Tx and ACL-R groups appear to be comparable (in location and severity), despite what appear to be significant differences in biomechanical joint stability.

As described by Deluzio et al., multidimensional datasets collected during biomechanical gait studies are large and complex [16]. Principal component analysis (PCA) is a data ordination technique that is used to reduce large numbers of potentially correlated variables to fewer uncorrelated variables that are linear combinations of the original variables (the principal components). PCA is useful to identify linear correlations among the original variables, and to identify those variables that account for the greatest portion of the total variance among subjects in the original data. PCA can be used on kinematic data as an exploratory analysis tool to describe features (kinematic patterns) within the data that can be interpreted in meaningful and more objective ways [17]. PCA has been used previously to identify and characterize subtle gait differences in persons with OA [18] or who are overweight [19]. PCA has also worked well in research designs with limited sample sizes [20].

The aim of this study was to compare post-surgical kinematic changes in the ACL-R model against those in a biomechanically stable control (CTRL) group and a biomechanically unstable ACL/MCL Tx group. Different from our previous approach that examined changes in isolated degrees of freedom (DOF) [13], here we describe the use of PCA to investigate each animal’s simultaneous change in the gait cycle from intact data at 4 and 20 weeks post-surgery using a combined measure of all DOFs, and examine each DOF at key points within the gait cycle in order to describe how each animal’s gait changed longitudinally after surgery. We tested the general prediction that if altered kinematics are responsible for the development of PTOA following surgery, then the magnitude of kinematic change of the ACL-R group would be different from that of a control group, and based on the similar amount of early PTOA development within surgical joints, would show the same magnitude of kinematic change as the ACL/MCL Tx group.

## Methods

### Surgical animal model

All surgeries and animal care & handling procedures were conducted following protocols approved by the University of Calgary Health Sciences Animal Care Committee. Fifteen skeletally mature female Suffolk-cross sheep (3 to 4 years old) were assigned to three groups (each *n* = 5): Idealized anterior cruciate ligament reconstruction (ACL-R), anterior cruciate ligament and medial collateral ligament transection (ACL/MCL Tx), or sex/age matched control (CTRL). Surgical methods for ACL-R and ACL/MCL Tx have been described previously [14, 21]. For all sheep, modified fracture plates were implanted on the lateral aspects of the right femur and tibia to facilitate the rigid attachment of posts that enabled 3D motion capture either optically (precision = 0.4°/0.4 mm) [9], or by way of an instrumented spatial linkage (ISL) (precision = 0.3°/0.3 mm) [22].

### Gross morphological score

The PTOA-like changes that occurred within the ACL-R and ACL/MCL Tx treatment groups have been described previously [10, 15]. Briefly, upon sacrifice the experimental and contralateral joints were graded for the presence of osteophytes (modified Grood [23]), cartilage damage (modified Drez [24]), and meniscal damage (modified Hellio La Graverand adapted from Adams [25]) and given a numerical score that represented the severity of change for each attribute. Scores for each of these attributes were summed to quantify the gross morphological score for the joint. To standardize the changes that occurred within each animal’s joint, the gross morphological score of the contralateral stifle joint was subtracted from the value of the experimental joint.

### Kinematic data collection

In vivo six degree of freedom (6-DOF) stifle joint kinematics were recorded at an uninjured baseline time-point (prior to surgical intervention), and then longitudinally at 4 and 20 weeks post-surgery. CTRL sheep followed the same protocol but did not undergo any stifle surgery. For each kinematic collection session, the removable posts were affixed to the previously implanted fracture plates. One hundred non-consecutive strides were measured while the sheep walked on a treadmill at 0.9 m/s. In the case of optical motion capture, data were collected at 120 Hz, filtered using a low-pass generalized cross-validation filter [26] with a cut-off frequency of 6 Hz and smoothed with a cubic spline. After the data for both the ACL/MCL Tx and ACL-R groups had been collected, a newer motion capture system was implemented to record the gait of the CTRL animals. CTRL group kinematics were collected using an instrumented spatial linkage (ISL) [27], and were filtered using a 4th-order low-pass Butterworth filter with a cut-off frequency of 30 Hz for data collected at 400 Hz. In addition to some increased accuracy in recording kinematics, the use of the ISL significantly reduced the amount of data processing required [22]. Anatomical coordinate systems were created post-mortem, the details of which are available elsewhere [9]. Data were normalized to 101 points, corresponding to 0–100% gait between successive hoofstrikes. Stifle kinematics were expressed in the Grood and Suntay joint coordinate system [28], and described as flexion/extension (FE), abduction/adduction (AA), internal/external rotation (IE), medial/lateral translation (ML), anterior/posterior translation (AP), inferior/superior translation (IS).

### Principal Component Analysis (PCA)

PCA was conducted on the average change-from-intact data from each post-surgical time-point (4 and 20 weeks post-surgery), and each DOF was composed of data from 6 predetermined locations within the gait cycle for: hoofstrike (HS; 0%), loading response (LR; 5 ± 1%), mid-stance (MS; 32 ± 1%), hoof-off (HO; 66 ± 1%), initial swing (ISW; 76 ± 1%), and terminal swing (TSW; 93 ± 1%). Thus, the PCA was conducted on the correlation matrix derived from a matrix that consisted of 26 rows, namely the 4 and 20 week post-surgical data from ACL/MCL Tx (*n* = 5), ACL-R (*n* = 4) and CTRL (*n* = 4) animals, and 36 columns, namely the observed change-from-intact at the 6 predefined gait points for each of the 6° of freedom (as described in the results section, one ACL-R and one CTRL animal were omitted as statistical outliers). PCA outputs were then used to characterize post-surgical differences between biomechanically unstable (ACL/MCL Tx) and presumably stable (ACL-R and CTRL) stifle kinematics.

### K-means clustering

An unguided clustering algorithm, k-means clustering, was used to validate the visual clusters of data apparent in the PCA scatterplots. Using principal component (PC) 1 and PC 2 as inputs, K-Means clustering analysis was employed in an attempt to distinguish 3 possible groups (k = 3) from the 4 and 20 week post-surgical data (*n* = 26): CTRL, ACL-R and ACL/MCL Tx. In order to account for the influence of potential auto-correlation on the k-means algorithm that stems from the closeness of post-surgical time points for each animal, two subsequent K-means clustering analyses were conducted (one k = 3, and one k = 2) using the average of the 4 and 20 week post-surgical time-points for each animal (*n* = 13). As the results reported by K-means clustering are sensitive to the random starting position within the data, results were replicated three times before interpreting the data.

### Calculation of absolute total change for each DOF

Average stride 6-DOF intact (pre-surgery) kinematics were determined from the 100 strides for each sheep, with an average value for each of 101 points in the gait cycle (each percent from 0 to 100%). For each DOF, an absolute total change parameter (ATC-P/DOF) matrix was created, for 4 and 20 week kinematics, by calculating the absolute difference from the average intact (baseline) kinematics for each point in the gait cycle (0–100%) for each of the 100 strides, then by summing the values within each row (stride, 0–100% gait cycle), and calculating the average of all 100 rows (strides). In this way, each sheep served as its own “internal” control while simultaneously preserving changes that may result from different surgical interventions. Each animal has 12 ATC-P values (6 DOFs x 2 post-surgical time points). Because the *absolute* change was calculated, the positive and negative differences from the average intact values were summed together rather than cancelling each other out.

## Results

Two animals were excluded as statistical outliers from the data set: one animal from the CTRL group and one animal from the ACL-R group. Each showed extremely high ATC-P values (+4 standard deviations from mean). These data were therefore excluded from the study, including the PCA analysis.

### Gross morphological score

The uninjured normal control joints received an average gross morphological score of zero, while the ACL-R and ACL/MCL Tx joints received comparable, and significantly elevated amounts of change when compared to the scores of the control joints. At the post-surgical time-point of 20 weeks, a significant amount of early PTOA-like changes had developed in the ACL-R and ACL/MCL Tx joints [10, 15].

### Principal component analyses

**Fig. 1:**
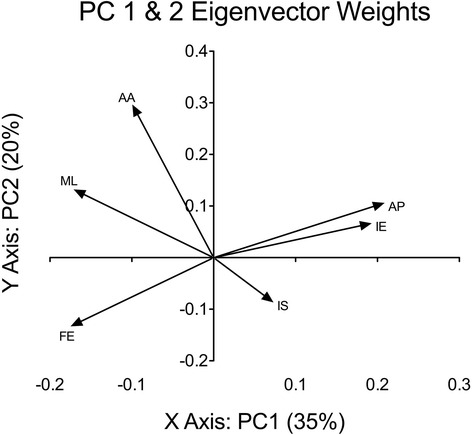
Principal Component Eigenvector Weights – Eigenvector weights are used to calculate the factor loadings that are used in the calculation of the principal component values (PC1 and PC2). Each of the 36 factors will influence the final PC value that will represent each ATC vector. The greater the eigenvector weight, the greater the factor loading value, the more influence that factor will have on the calculation of the principal component. Within this figure, each of the eigenvector weights that represent the 6 gait points within a DOF were averaged to provide a single DOF value. Arrows represent how a positive original variable would load (a negative variable would load in the opposite direction). For example, in calculating the PC values for an animal, if that animal had a positive change from intact in FE, its score for PC1 would be positive (in quadrant III), if that same animal showed a negative change from intact in FE, its PC1 score would be negative in quadrant I

PCA on the change from intact (baseline) data at both the 4 and 20 week post-surgical time points indicated that the first three components accounted for ~73% of the variance in the six DOFs at the two post-surgical time-points (Table 1). Not surprisingly, PCA also indicated that the six points in the gait cycle were highly correlated within a given DOF (average correlation = 0.76, range 0.66–0.89) (Additional file 1 Figure S1). Therefore, the six points in the gait cycle were averaged within each DOF in the following interpretations of the PC loadings. Figure 1 summarizes the eigenvector weights, which were used to calculate the factor loadings for the first three PCs (see also Table 1 for correlation coefficients). The factor loading data suggested that anterior-posterior translation (AP) and internal-external rotation (IE) of the stifle were strongly positively correlated with PC1, while flexion-extension (FE) and medio-lateral translation (ML) were negatively correlated with this PC. Abduction/adduction (AA) was strongly positively correlated with PC2, while inferior-superior translation (IS) loaded most strongly on PC3.

**Table 1:** Principal Component Analysis Summary Table

Components	Eigenvalue	Proportion	Cumulative	Average Factor Loading					
				AA	AP	FE	IE	IS	ML
PC 1	12.54	35%	35%	−0.4	0.7	-0.6	0.7	0.3	-0.6
PC 2	7.08	20%	55%	0.8	0.3	−0.4	0.2	−0.2	0.4
PC 3	6.57	20%	55%	0.3	0.4	0.3	−0.5	0.6	−0.1

**Fig. 2:**
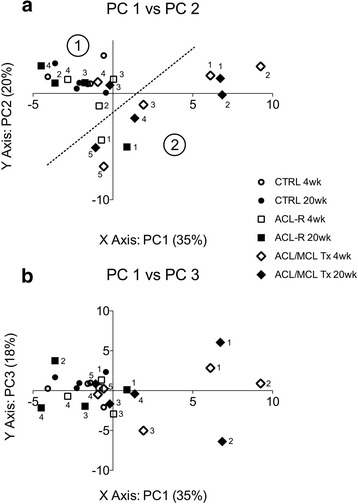
Principal Component Analysis Scatterplots (combined 4weeks and 20week change from intact data). **a** The scatterplot of PC1 vs PC2 data reveals that CTRL, ACL-R animals at 4 and 20 weeks post-surgery group together (Group 1), and data for the ACL/MCL Tx group and make up the second group (Group 2) - divided by the arbitrarily placed dashed line. Of note, one ACL-R animal grouped with the ACL/MCL Tx animals. **b** The scatterplot of PC1 vs PC3 data further reveals some of the variability in the ACL/MCL Tx group with PC3 values ranging from −5 to 5. Additionally, one ACL-R data point also showed a greater loading on PC3 than the majority of CTRL and ACL-R animals that grouped more closely together

The visual representation of PC 1 and 2 revealed an apparent separation of data points into two distinct groups (Fig. 2a, dotted line). As shown in Fig. 2a, the CTRL 4 and 20 week change from intact data clustered closely together in quadrant II with the majority of the ACL-R data. Two ACL/MCL Tx animals grouped closely with the CTRL and ACL-R animals in quadrant II (ACL/MCL Tx #3 at 4 weeks post-surgery, and ACL/MCL Tx #4 at 20 weeks post-surgery). At the respective 20 and 4 week post-surgical time points for these animals, they were placed in quadrant IV and clustered with the other ACL/MCL Tx animals. Two ACL/MCL Tx animals showed change from intact (at both post-surgical time points) predominantly in quadrant I, associated with positive change from intact in AP and IE (increased anterior tibial translation and internal rotation), and negative change-from-intact in FE and ML (increased knee flexion and more medially positioned tibia throughout the gait cycle), while one animal predominantly in quadrant III, associated with positive changes in AA (increased tibial adduction throughout the gait cycle).

The scatterplot of PC1 vs PC3 (Fig. 2b) visualizes the data that correlated with PC3, which were predominantly positively correlated to IS (Table 1). PC3 data suggested there is both inter- and intra-animal variation within the post-surgical change from intact values of IS within the ACL/MCL Tx group.

Interestingly, one ACL-R animal (ACL-R #1) clustered with the majority of ACL/MCL Tx animals at 4 and 20 weeks post-surgery (Fig. 2a quadrants III and IV respectively), suggesting substantial, and potentially pathological, gait changes in this sheep despite the reconstructive surgery.

### K-means clustering

Two groups of data were apparent within the graphical representation of PCA output, depicted by the dashed line in Fig. 2a. These visual groupings were investigated with an unguided clustering algorithm (K-means clustering). As summarized in Table 2-A, it was found that the ACL/MCL Tx group was indeed distinguishable from the normal and ACL-R groups. Eighty percent of the ACL/MCL Tx group were identified correctly by K-means, with two ACL/MCL Tx animals (#3 at 20 weeks and #4 at 4 weeks post-surgery) grouping with the CTRL/ACL-R cluster. The algorithm was not able to distinguish between the change from intact gait of CTRL and ACL-R animals. ACL-R animal #1 was grouped with the ACL/MCL Tx animals by K-Means. These results were replicated when the 4 and 20 week post-surgical data were averaged, and k-means clustering was conducted to classify the data into three groups (Table 2-B), and two groups (Table 2-C).

**Table 2:** Output from K-Means Clustering

A.	*n* = 26		
	Group 1	Group 2	Group 3
CTRL	0	6	2
ACL-R	2	3	3
ACL/MCL Tx	8	2	0
B.	*n* = 13		
	Group 1	Group 2	Group 3
CTRL	0	3	1
ACL-R	1	3	0
ACL/MCL Tx	5	0	0
C.	*n* = 13		
	Group 1	Group 2	
CTRL	0	4	
ACL-R	1	3	
ACL/MCL Tx	5	0	

### Absolute total change parameter for each DOF

**Fig. 3:**
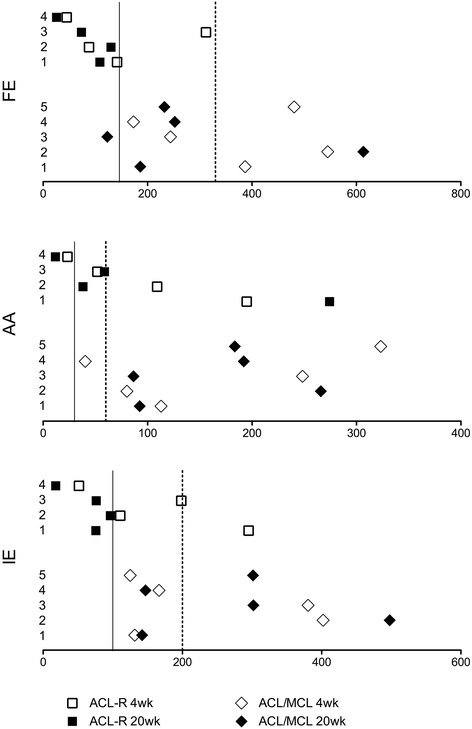
Absolute total change parameter (ACT-P) values for Rotations of ACL-R and ACL/MCL Tx animals. Each animal is numbered to correspond to the PCA scatterplots in Fig. 2. Values represent the absolute change from intact at the 4 and 20 week post-surgical time-points in the following DOFs: FE = Flexion/Extension, AA = Abduction/Adduction, IE = Internal/External. Solid line = mean normal control value. Dashed line = 1 standard deviation from normal control mean

**Fig. 4:**
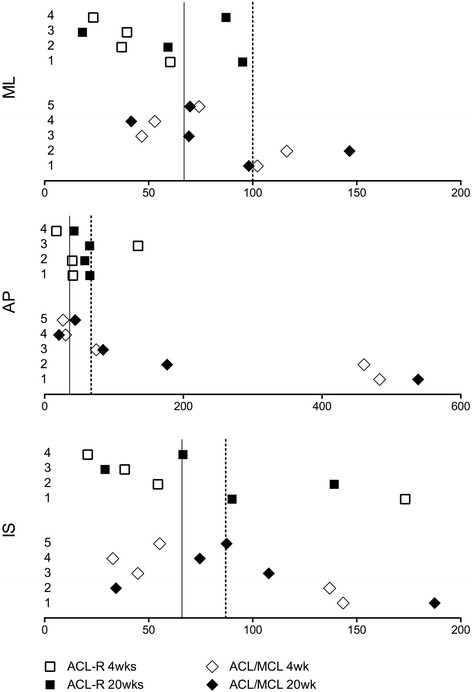
Absolute total change parameter (ACT-P) values for Translations of ACL-R and ACL/MCL Tx animals. Each animal is numbered to correspond to the PCA scatterplots in Fig. 2. Values represent the absolute change from intact at the 4 and 20 week post-surgical time-points in the following DOFs: ML = Medial/Lateral, AP = Anterior/Postieror, IS = Inferior/Superior. Solid line = mean normal control value. Dashed line = 1 standard deviation from normal control mean

For each animal, the ATC-Ps for each DOF were calculated for each post-surgical time-point (Fig. 3 - Rotations, and Fig. 4 - Translations) and presented in relation to the mean and standard deviation of the CTRL group.

Kinematic change within animals of the ACL-R group generally resided within the normal variation of the normal control group; however as identified by PCA, ACL-R animal #1 showed a greater magnitude of change from intact in the coronal plane motion at 4 and 20 weeks post-surgery, and internal/external rotation at 4 weeks post-surgery (Fig. 3). Furthermore, an increase in the magnitude of inferior/superior translation at 4 and 20 weeks post-surgery was also noted in this animal.

Kinematic change from intact in animals of the ACL/MCL Tx group was more variable than that of the ACL-R group, shown as a larger spread in ATC-P (x-axis) values in Figs. 3 and 4. With the exception of ACL/MCL Tx animal #2, all animals were within the normal range of Flexion/Extension by 20 weeks post-surgery. Conversely, all ACL/MCL Tx animals had exceeded the normal ranges of Abduction/Adduction by 20 weeks post-surgery.

## Discussion

The aim of this study was to compare post-surgical kinematic changes in the ACL-R model against those of a biomechanically stable control group (CTRL), and a biomechanically unstable ACL/MCL Tx group, to determine whether kinematic change could be driving the early PTOA-like changes in the ACL-R group that we reported previously [15]. This was accomplished by using a data reduction technique (PCA) to facilitate comparisons of individual animals by removing the complexity of the kinematic data set while maintaining the majority of the individual variation within the data set. PCA was used to describe the kinematic change from intact values simultaneously at 6 key points in the gait cycle and each DOF, and then these results were interpreted with the aid of a new analysis that examined the absolute kinematic total change from intact that occurred throughout the gait cycle. This new approach yielded information about the gait cycle in its entirety.

There are three key findings from our analysis: 1) kinematic change from intact data of most ACL-R animals clustered with that of the normal control group, and this cluster was distinguishable from the cluster that consists of predominately ACL/MCL Tx kinematic change from intact data; 2) kinematic change from intact data of the ACL/MCL Tx group at 4 and 20 weeks post-surgery was mainly defined by negative changes in abduction/adduction (i.e., increased stifle adduction), positive changes in anterior/posterior translation (i.e., increased anterior tibial position), and an increase the inferior/superior position (i.e., more superiorly positioned tibia); and 3) the kinematic change from intact data from 6 points throughout the entire gait cycle were equally informative when interpreting this type and format of data, with the caveat that each DOF was considered in relation to the other DOFs simultaneously. Therefore, studies on kinematic change between treatment groups that focus on the investigation of single points in the gait cycle may be justified in reducing their data to these points, but only if they consider all DOFs simultaneously.

PCA on the change-from-intact data (with direction, not absolute values) enabled the visual identification of two clusters of animals (Fig. 2a). The first cluster consisted of predominantly CTRL and ACL-R animals, while the second cluster consisted of predominantly ACL/MCL Tx animals (and appeared to be more variable than the first cluster). As the kinematic change from intact data of ACL-R and CTRL animals clustered together, these results did not support our hypothesis, and suggested that idealized ACL-R surgery does not meaningfully alter kinematics in most sheep as compared to kinematics of non-operated CTRL sheep.

By interrogating the kinematic data as the change-from-intact for each sheep, we preserved much of the individual variation in kinematics that is known to exist in this animal model [9], but standardized the data in a way that facilitated treatment group comparisons. Furthermore, as each of the 6 points in the gait cycle were correlated for each DOF, these data suggest that these points in the gait cycle are equally informative when analyzing gait when each DOF (motions that are also likely coupled [18]) is simultaneously considered in the analysis. Finally, we described the total kinematic change in each DOF by presenting the absolute changes in the gait cycle for each DOF as the ATC-Ps. All data points that span a complete gait cycle were included in this approach, meaning that the ATC-Ps for each DOF are, in a sense, a knee joint global change parameter.

Of note, it was observed that CTRL ATC-Ps were non-zero, suggesting that normal non-pathological gait is variable, and indicating that longitudinal changes may occur even in the CTRL sheep due to the experimental environment. On average, the biomechanically unstable ACL/MCL Tx ATC-P values were nearly 50% greater than those of CTRL, which is likely a biologically relevant magnitude given the OA-like changes reported in this injury model [10]. In the ACL-R group, the average ATC-P values were greater than those of CTRL by approximately 2–4%. A non-significant elevation that is within the variation of the CTRL group.

Previously, we have reported changes in 6 DOF kinematics for both ACL/MCL Tx and ACL-R sheep [11]. While longitudinal 6 DOF ACL/MCL Tx kinematic changes were more pronounced in these previous analyses, ACL-R led to subtle internal tibial rotation during weight acceptance, anterior tibial translation at mid-stance, and a more inferior tibial position at hoof-off compared to sham-operated control [13]. In this previous study, we investigated the change from intact data at single points within the gait cycle, and within each DOF separately, an analysis that required multiple statistical comparisons. Using the new methodology described in the present study, we have limited the error that can be attributed to multiple statistical comparisons and we feel that we have provided a more accurate analysis of the global kinematic change of the stifle joint. Furthermore, although the ACL-R changes were statistically significant compared to sham in this previous analysis, arthrotomy alone may also affect longitudinal 6 DOF kinematics [11]. Group differences between ACL-R and sham kinematics we reported previously could be attributed to the sham kinematics changing in the opposite direction to those of the ACL-R sheep.

While expressing kinematic change in each DOF using the absolute change from intact simplified the complexity of the data, use of the ATC-P provides less information about the nature of that change, i.e., directional change in specific degrees of freedom. However, the use of ATC-P in combination with PCA as a first step towards determining where differences lie among (and within) multiple experimental groups and at key points of the gait cycle is a powerful tool. The ATC-P analysis aided in the interpretation of the PCA by showing the variation, within and between treatment groups, that was used to construct the PCs. Furthermore, by investigating the absolute change in kinematics by way of the ATC-P – which eliminates the confounding effect of direction – perhaps we have increased the sensitivity to detect true between-group differences assuming that any changes from baseline are of equal importance clinically. Our collective results in these injury models suggest that in addition to an intact (pre-surgical) control, studies that measure gait over time should also consider having a normal, time related, experimental control.

In the development of PTOA/OA, the stance portion of the gait cycle, in which the cartilage surfaces are loaded, is likely the most important to investigate the change in kinematics that could be attributed to (or lead to) cartilage damage. As we have shown that when each DOF is considered simultaneously the entire gait cycle (extrapolating from the 6 gait points we investigated via PCA) is of equal importance in the investigation of kinematic changes between treatment groups, perhaps this finding corroborates studies that use stance phase data as a surrogate for change of the entire gait cycle.

As with many large animal studies, sample size is a limitation in this study. Only 3/5 ACL-R animals were shown to be in the PCA cluster with controls. One animal had been omitted and one was in the ACL/MCL-Tx cluster. Therefore, it is possible that the lack of differences between control and ACL-R could be due to a lack of statistical power from the small number of samples. A greater number of animals within each treatment group would enhance the interpretations drawn from the PCA. For example, with the addition of more animals into each group, it may be possible to refine the interpretations of each PC in describing biologically relevant kinematic features that account for less of the variance. When principal components begin describing very small proportions of the variance present in the data set (<5%), the accurate interpretation of the features they describe can become increasingly complicated [17]. In our model, larger sample sizes may facilitate future investigation of these PCs. Additionally, while the work presented in this paper does not address the direct mechanisms of PTOA development, we have previously explored change in path length, contact velocity, and distance between subchondral surfaces in the ACL/MCL Tx model that correlates to the pathological changes present in the joint [12, 29].

## Conclusion

In conclusion, we have investigated the kinematic change within an ovine model of idealized ACL reconstruction (ACL-R), against that of stable (CTRL) and unstable (ACL/MCL Tx) joints. In conjunction with previous studies of these animals, we have found that despite having comparable early PTOA-like changes between ACL-R and ACL/MCLx groups, only the ACL/MCL Tx group appeared to differ from the CTRL group in the deviation from baseline (pre-injury) kinematics. We have reinvestigated ACL-R and ACL/MCL Tx in the context of a newly acquired age-matched control group (CTRL) by creating and investigating the absolute total change parameter (ATC-P), an approach that captures the absolute magnitude that each animal’s post-surgical joint kinematics deviates from its own average normal kinematics throughout the entire gait cycle, and over 100 strides. We used this analysis to complement PCA that simultaneously investigated 6 key gait points within each DOF. Results indicated that ACL/MCL Tx created significantly greater kinematic deviation relative to ACL-R and CTRL, whereas ACL-R maintained joint kinematics similar to CTRL. PCA revealed that data from CTRL and ACL-R animals clustered together, while data from ACL/MCL Tx animals clustered together and were more variable between animals. This work suggests that the early PTOA like changes following idealized ACL-R may not be related to overt abnormal gross joint kinematics, but instead may be driven mainly by biological (inflammatory) mechanisms that alter interactions at the cartilage surface within the first 20 weeks post-operatively.

## Additional file

Additional file 1: Figure S1. Full factor loading plot. Points labeled by DOF_Gait point. This figure shows the full factor loadings from the principal components analysis. PC 1 (35%) is the X axis, PC 2 (20%) is the Y axis. Each factor is labeled by [DOF]_[Location within the gait cycle]. For example, in quadrant 2, ml_ho represents the factor loading for [Medial/Lateral]_[Hoof Off]. (JPG 1433 kb)

## Abbreviations

AA: Abduction/Adduction
ACL: Anterior Cruciate Ligament
ACL-R: Anterior Cruciate Ligament - Reconstruction
ACL/MCL Tx: Anterior Cruciate Ligament/Medial Collateral Ligament
AP: Anterior/Posterior
ATC-P: Absolute total change parameter
CTRL: Control
DOFs: Degrees of Freedom
FE: Flexion/Extension
HO: Hoof off
HS: Hoof Strike
Hz: Hertz
IE: Internal/External
IS: Inferior/Superior
ISL: Instrumented spatial linkage
ISW: Initial Swing
LR: Load Response
m/s: Meters/second
MCL: Medial Collateral Ligament
ML: Medial/Lateral
mm: Millimeter
MS: Mid-stance
PC: Principal Component
PC1: Principal Component 1
PC2: Principal Component 2
PC3: Principal Component 3
PCA: Principal Component Analyses
PTOA: Post-Traumatic Osteoarthritis
TSW: Terminal Swing
