# Supplementary figures and images for: Analysis of change in gait in the ovine stifle: normal, injured, and anterior cruciate ligament reconstructed
Source: BMC Musculoskelet Disord. 2017 May 23;18:212. doi: 10.1186/s12891-017-1576-3 (PMC5442660; doi:10.1186/s12891-017-1576-3)

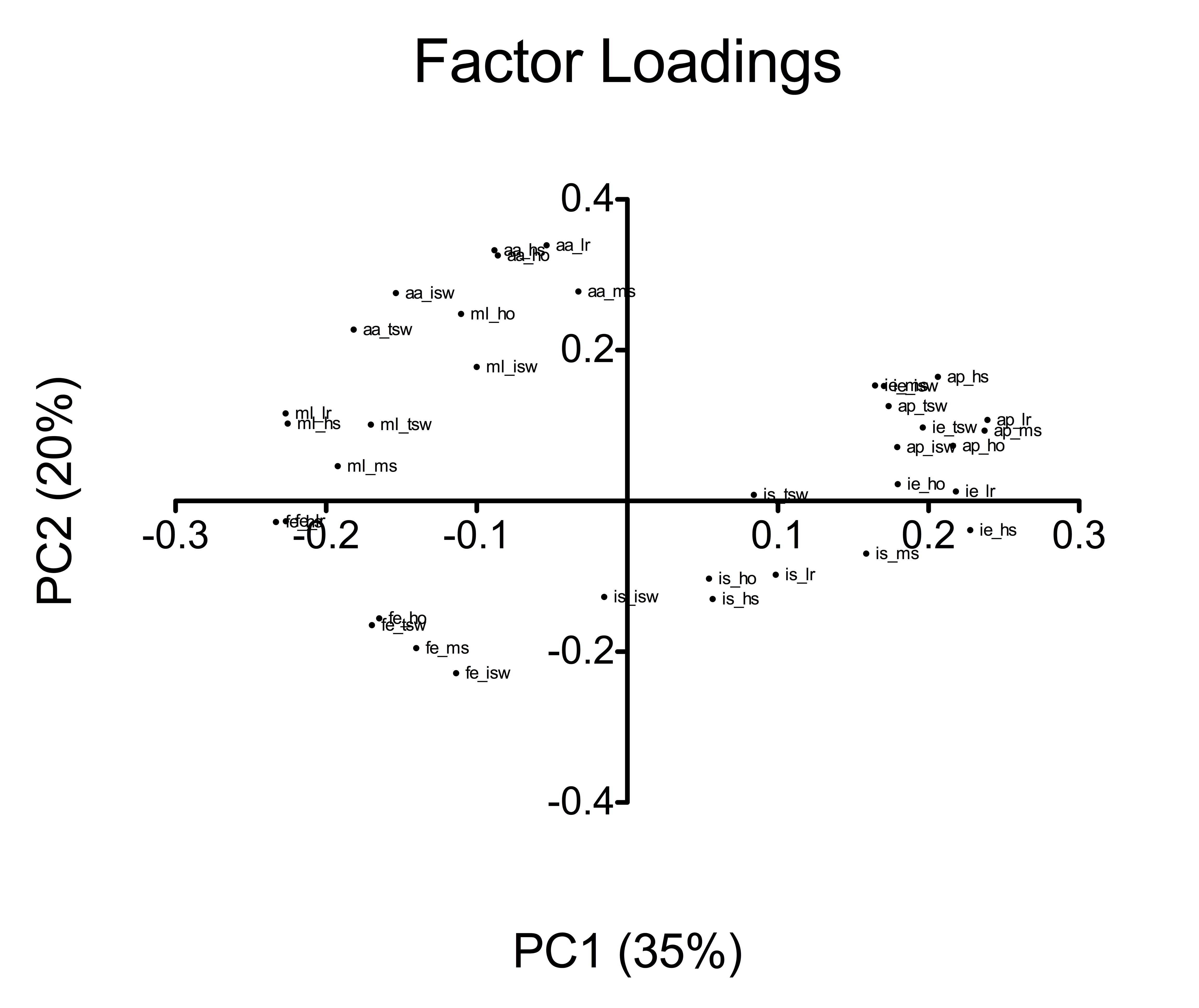

Supplement: Additional file 1: Figure S1. — Full factor loading plot. Points labeled by DOF_Gait point. This figure shows the full factor loadings from the principal components analysis. PC 1 (35%) is the X axis, PC 2 (20%) is the Y axis. Each factor is labeled by [DOF]_[Location within the gait cycle]. For example, in quadrant 2, ml_ho represents the factor loading for [Medial/Lateral]_[Hoof Off]. (JPG 1433 kb) [file 12891_2017_1576_MOESM1_ESM.jpg]
